# A pilot study: tail tip lesions in dairy cows – an unnoticed animal welfare issue?

**DOI:** 10.5194/aab-67-271-2024

**Published:** 2024-06-25

**Authors:** Prisca V. Kremer-Rücker, Kathrin M. Abel, Lea M. Lorenz, Christine Schmidt, Mirjam Lechner, Kim F. Schubert, Amalie A. Köhler, Saskia Meier, Armin M. Scholz

**Affiliations:** 1 Animal Health and Welfare in Livestock Breeding, Faculty of Agriculture, Food and Nutrition, Hochschule Weihenstephan-Triesdorf University of Applied Sciences, 91746 Weidenbach-Triesdorf, Germany; 2 Unabhängige Erzeugergemeinschaft Hohenlohe Franken, 97996 Niederstetten-Adolzhausen, Germany; 3 Livestock Center Oberschleissheim, Veterinary Faculty, University of Munich, Oberschleißheim, Germany

## Abstract

The welfare of dairy cows is becoming increasingly important. While diseases like mastitis and lameness are common ailments, injuries to the tail tip go largely unnoticed. This study aimed to investigate whether tail tip lesions, which are mostly described in beef cattle, also occurred on n=5 dairy farms, along with determining what type and at what frequency. The study consisted of two phases. During the first part of the study, tail tips of 78 dairy cows were examined over a period of 6 months; based on these results, we developed a training card on tail tip lesions in dairy cows, which was used in part two of the study to train further examiners to inspect four more flocks. In total, we collected n=3587 tail records from n=513 Holstein and n=128 Simmental dairy cows. The overall frequency regarding all types of lesions ranged between 84.0 % ( ±2.0 ) and 94.1 % ( ±1.8 ) in Holstein herds and between 97.0 % ( ±2.2 ) and 99.0 % ( ±2.2 ) in Simmental herds. To our knowledge, this is the first investigation of tail tip lesions in German dairy cows. We concluded that tail tip lesions might be a frequent yet unnoticed condition in German dairy cows.

## 1 Introduction

Animal health and welfare in dairy cattle are increasingly becoming a focus of both farmers and consumers across countries (Special Eurobarometer 442: Attitudes of Europeans towards Animal Welfare – Data Europa EU, 2023; Blanco-Penedo et al., 2020; Bauman et al., 2016). Attention has mainly been paid to the most common causes of involuntary culling and, thus, diseases of the reproductive tract, the udder, and the limbs (Chiumia et al., 2013). With regard to inappropriate housing conditions, lesions on the hock, tarsal joint, or neck are often investigated (Fulwider et al., 2007; Kielland et al., 2010; Jewell et al., 2019). Research focused on tail lesions has come to be part of the work focused on intensively housed fattening cattle (Drolia et al., 1991; Schrader et al., 2001; Kordowitzki, 2015). More often discussed in relation to the tail tips of dairy cows is tail docking, which is still practiced in some regions of the world to improve milking hygiene and the occupational safety of milking personnel in relation to the transmission of leptospirosis (Barnett et al., 1999; AVMA, 2014). However, as tail docking is associated with pain and suffering for the animal, regular trimming of the switch is seen as an alternative (Stull et al., 2002). This attention to the tail might be the reason why there is growing evidence indicating a high prevalence of tail lesions in dairy cows globally (Moorman et al., 2018; Crossley et al., 2022; Moono et al., 2022). In principle, there is a moral obligation to minimize pain in farm animals. Integument lesions at the tail tip constitute a painful health issue and can result in tail tip necrosis and part loss. In beef cattle, the prevalence of lesions was reported to increase when animals were housed on slatted flooring (Drolia et al., 1991; Schrader et al., 2001). Also, a lower rumen pH was found in bulls affected by tail tip necrosis compared to unaffected bulls, indicating a possible association between (sub-) acute rumen acidosis and tail tip necrosis (Kordowitzki, 2015). Thus, high concentration ratios that substantially lower the pH level in the rumen might be risk factors for tail tip inflammation and necrosis (Abdela, 2016). The occurrence of tail tip inflammation and necrosis is also often described as being related to high stocking density (Kordowitzki, 2015; Madsen and Nielsen, 1985; Kroll et al., 2014), season (Schrader et al., 2001; George et al., 1970), tail twisting during handling of the animals (Zurbrigg et al., 2005; Olsen et al., 2023), or social interactions which may affect the tail (Tucker et al., 2015). Even cases of tail biting, at least in calves, are reported in the literature (Van der Mei, 1986; Millar and Kenward, 2015). Besides fattening cattle, tail lesions are known to occur in many different species (Barakat et al., 1960; Barthold et al., 2016; Bridle and Littlewood, 1998). In pigs, a syndrome called “swine inflammation and necrosis syndrome” (SINS) has been described, which causes inflammation and necrosis of the tail (tip and base), ears, and teats and laminitis of the claws (Reiner et al., 2021). In dairy cows, however, the literature regarding tail tip inflammation is scarce, although research in this area could help to identify possible risk factors and assess the impact on cow welfare.

Considering the fact that inflammation and/or necrosis of the tail have been described in many species, including cattle, while there is little research on this subject in dairy cows, the aims of this study were to evaluate (1) whether tail tip lesions occur in dairy cattle, (2) what type of lesions are found in dairy cattle, and (3) how often lesions occur.

## 2 Material and methods

### 2.1 Animals and data collection

This study was divided into two sections. During part one of the study, data collection was conducted over a period of 6 months, starting in December 2019. The herd was a randomly selected German dairy herd of 80 Holstein Friesian (HOL) cows (herd A), where the hair of the switches was routinely shorn. All milking cows were included in the data collection. Only dry cows and animals with apparent partial tail amputation due to other causes were excluded. A total of n=78 different cows were examined during the data collection period. The tails were examined every 2 weeks for any kind of tail lesions. The number of cows examined varied between n=67 and n=72 on each examination date.

**Figure 1:**
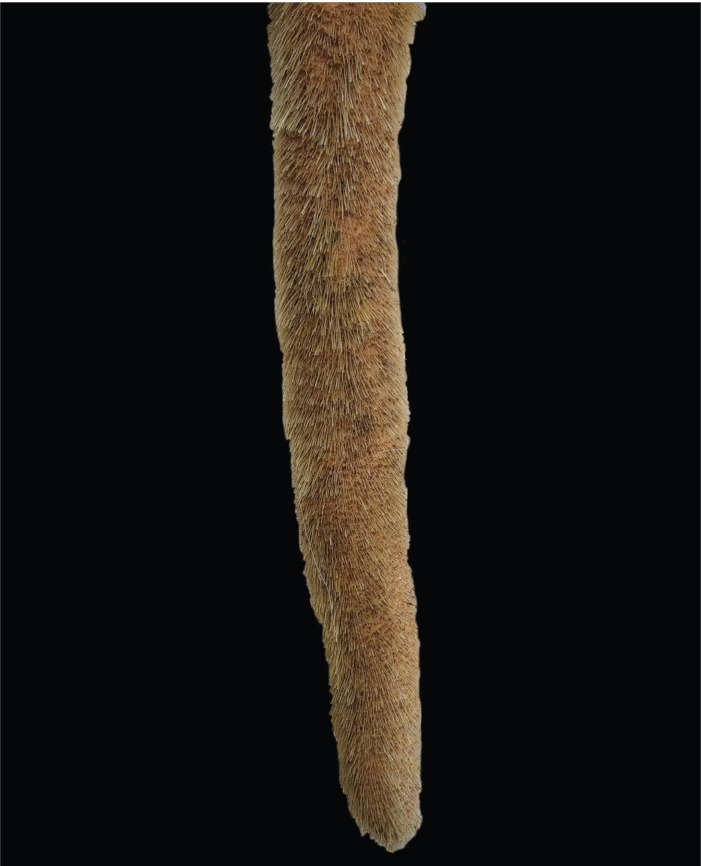
Unaltered tail tip of a dairy cow: fully covered by hair, no lesions, and tapering towards the tip.

Data collection was performed as follows: after shaving the distal part of the tail, including the switch, using a hand clipper, the tails were washed with warm water and mild soap. The tails were then inspected and palpated by a single animal scientist. An unaltered (physiological) distal part of the tail was defined as slender, tapering towards the tip, straight, completely covered by hair, free of lesions, and covered by intact skin (Fig. 1).

**Figure 2:**
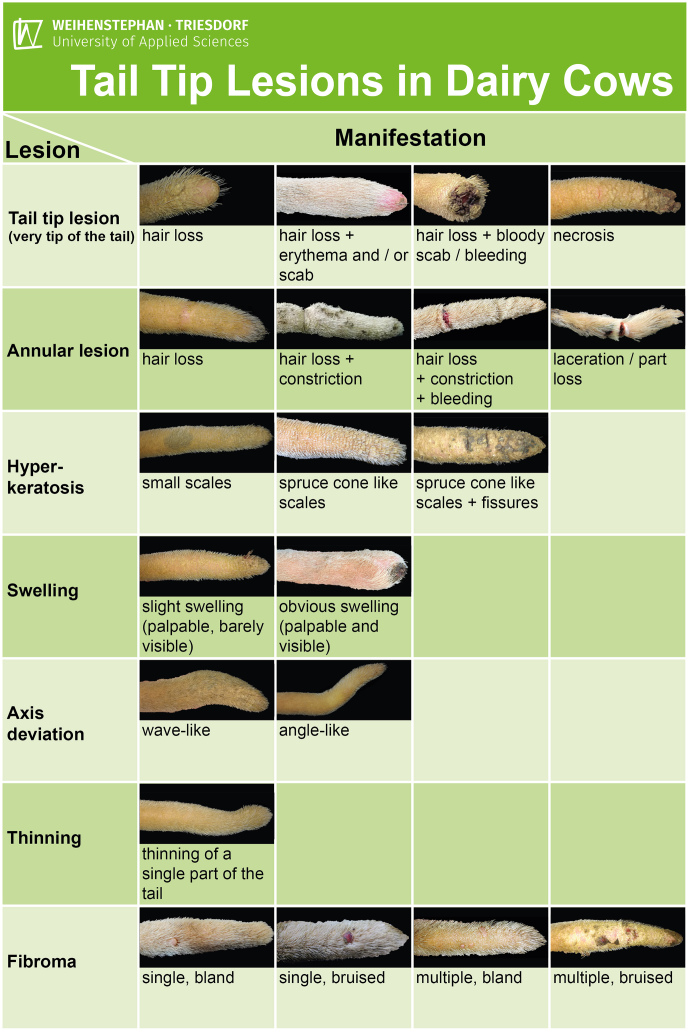
Training card depicting tail tip lesions in dairy cows, differentiating seven different types of lesions and their observed manifestations at the spineless part of the tail.

All findings differing from an unaltered tail were recorded, photographed with a mobile phone (Huawei P9 lite, Huawei Technologies Co., Ltd., Shenzhen, China), and described in detail. In total, n=833 tail examinations originating from 78 cows were assessed. Based on these observations, seven different types of lesions located at the tail tip were identified. A training card was developed to provide an overview of the tail tip lesions observed (Fig. 2) to help train other examiners.

During part two of the study, cows from herd A and cows originating from four other randomly selected German dairy herds (herds B, C, D, and E), in which the switches of the cows were routinely sheared, were examined (Table 1).

**Table 1:** Overview regarding the observed herds and animals during part two of the study.

Herd	A	B	C	D	E	?
Breed	HOL ${}^{1}$	HOL	HOL	SIM ${}^{2}$	SIM	2
Average herd size (cows)	80	1.00	300	70	90	
Average milk yield per year (kg)	10 149	12 383	10 946	9445	7199	
Observed animals ( n )	80	215	218	67	61	641
Observation period (months)	8	8	7	6	6	
Examinations (interval (weeks)/ n )	4/9	4/8	2/10	3/8	3/8	
Number of observations ( n )	573	1716	380	495	423	3587
Flooring	slatted	slatted	solid	slatted	solid	
	concrete	rubber	concrete	concrete	concrete	
Automatic scraper system	no	yes	yes	no	yes	
Cubicles	rubber	straw	straw	rubber	rubber	
	mats	manure	manure	mats	mats	
		mix/	mix			
		water bedding				

${}^{1}$ Holstein Friesian. ${}^{2}$ Simmental.

Cows from herd A were examined by the same person as in part one of the study. Four more examiners were trained based on the abovementioned training card and subsequently performed the examination of tails in herds B, C, D, and E. The training of the examiners consisted of a theoretical part of approximately 45 min and a practical on-farm unit of approximately 90 min. Additionally, all persons were taught to shave the distal third of the tails very carefully in order not to cut into the skin. The examination was carried out in the same way as in the first part of the study, and only the washing of the tail tips was omitted. Examiners were also asked to record, photograph, and describe any type of lesion not shown on the training card.

The cows included in this part of the study originated from three HOL herds, with average herd sizes of n=80 , 1300, and 300 (herd A, B, C, respectively), and two Simmental (SIM) herds, with 70 (herd D) and 90 cows (herd E). All cows were housed in loose housing systems. No pasture was provided. Herd size, milk yield, housing conditions, and numbers of examinations are shown in Table 1. Tail tip examination included either all cows or a sample of the herd. Animals with already obviously (partially) amputated tails were excluded from data collection in advance. Likewise, animals whose tails had to be amputated during data collection for medical reasons due to injuries to the tip of the tail were also excluded from further investigations. As the data collection took place in the field and not under experimental conditions, only the animals made available per herd by the farmer could be studied. In herds A, D, and E, these were almost all milking animals, and in herd B, there was access to cows that had recently calved and could be followed in the high-performance group and beyond. In herd C, there was access to a specific compartment of the barn, and the cows kept there represented a variable group.

Data collection was performed from April to November 2021 (herd A, B), from May to November 2021 (herd C), and from June to November 2021 (herd D, E). In total, n=3587 tail examinations originating from 641 different cows were performed during part two of the study. All findings obtained during part one of the study, with the exception of a single axis deviation combined with palpable bony callus, were located at the spineless part of the tail. Therefore, the examination during part two of the study focused only on the distal quarter, including the spineless part of the tail.

### 2.2 Data analysis

Data preparation and analysis were performed using R version 4.1.2 (R Core Team). To calculate the frequency of tail tip lesions, all observations were encoded in binary, with 0 indicating the absence of tail lesions and 1 indicating the occurrence of any kind of lesion, following the approach described by Lin and Chu (2020) and Salinas Ruiz et al. (2023). The observation of one or more tail tip lesions therefore counted as an altered tail tip, encoded with 1. A tail tip considered to be unaltered was encoded as 0. As cows were examined repeatedly (Table 1), all observations resulting from the respective time periods were taken together per herd, per lesion, and per breed.

During part one of the study, a simple percentage evaluation was performed for the analysis of the frequency of altered tail tips. During part two, the frequencies of tail tip lesions per breed were tested in a linear mixed model (lmer function) using the following formula:

$$y_{ijk}=\mu +B_{i}+c_{j}+\varepsilon_{ijk},$$

where $y_{ijk}$ is the frequency of binary-encoded tail tip alterations ( n=6 ), μ= is the expected value of y , $B_{i}$ is the fixed effect of breed ( i= HOL, SIM), $c_{j}$ is the random effect of the cow ( j=1 , …, 641), and $\varepsilon_{ij}$ is the random effect of the residuals.

The frequencies of tail tip lesions per herd were tested in a linear mixed model (lmer function) using the following formula:

$$y_{ijk}=\mu +H_{i}+c_{j}+\varepsilon_{ijk},$$

where $y_{ijk}$ is the frequency of binary-encoded tail tip alterations ( n=6 ), μ= is the expected value of y , $H_{i}$ is the fixed effect of herd ( i= A, B, C, D, E), $c_{j}$ is the random effect of the cow ( j=1 , …, 641), and $\varepsilon_{ijk}$ is the random effect of the residuals.

We then used the emmeans function in order to obtain least-square means (LSMs) and their standard errors (SEs) of estimation, which were multiplied by 100 and therefore expressed in percent (%).

## 3 Results

### 3.1 Observed tail tip lesions

During part one of the study, n=833 tail tip records originating from 78 cows were collected. Within this data set, the percentage of affected tail tip records showing at least one lesion was 72.27 %.

**Figure 3:**
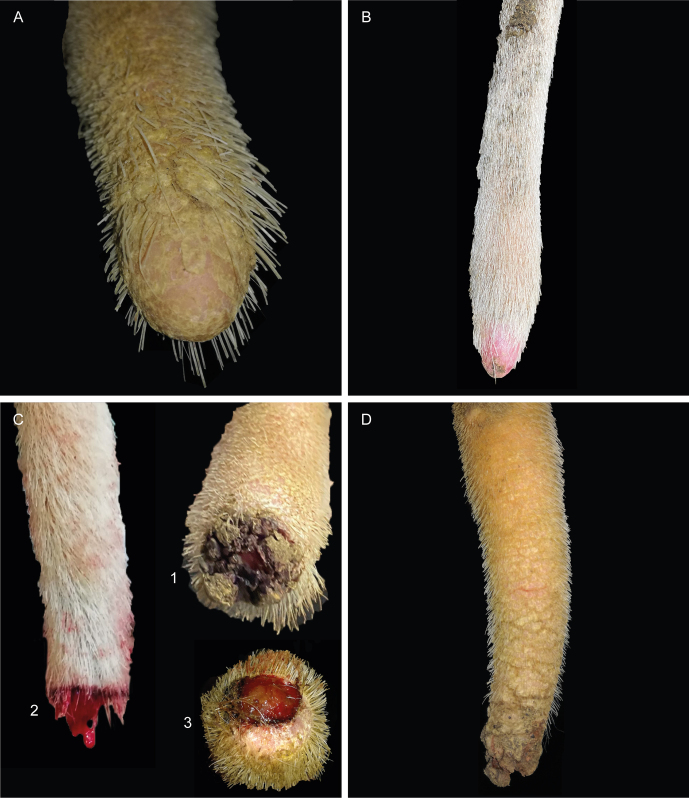
Tail tip lesion (very tip of the tail). **(a)** Airless tip; **(b)** erythema of the tip, covered by a small scab; **(c)** tail tips showing bloody scabs (1), bleeding (2), or granulation tissue (3); **(d)** necrosis.

**Figure 4:**
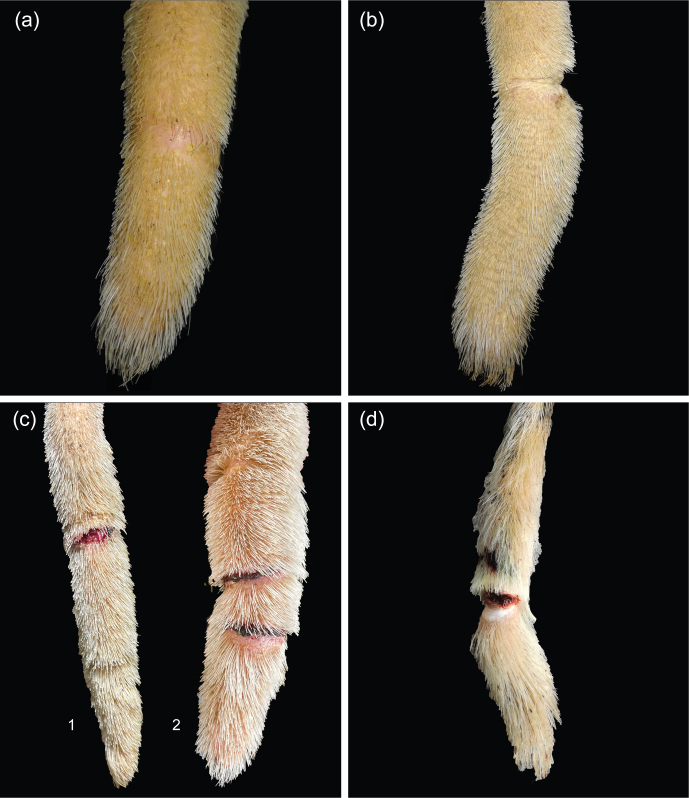
**(a)** Annular lesion, hairless ring; **(b)** annular constriction; **(c)** bleeding constriction accompanied by two hairless rings (1) and multiple annular constrictions, bland or covered by scabs (2); **(d)** laceration of the tail tip, beginning part loss.

**Figure 5:**
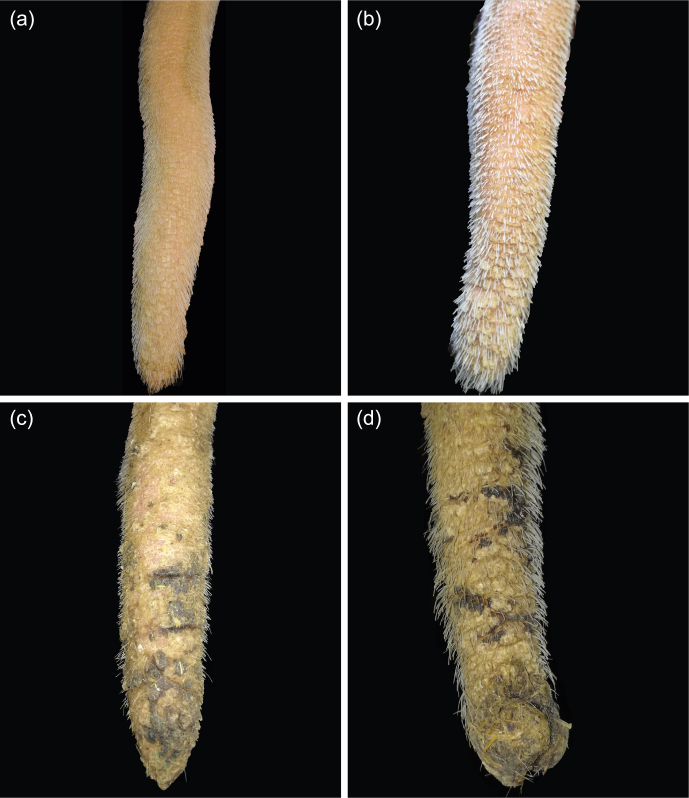
Lesion: hyperkeratosis. **(a)** Small scales, **(b)** spruce-cone-like scales, **(c)** small scales and fissures, **(d)** spruce-cone-like scales and fissures.

**Figure 6:**
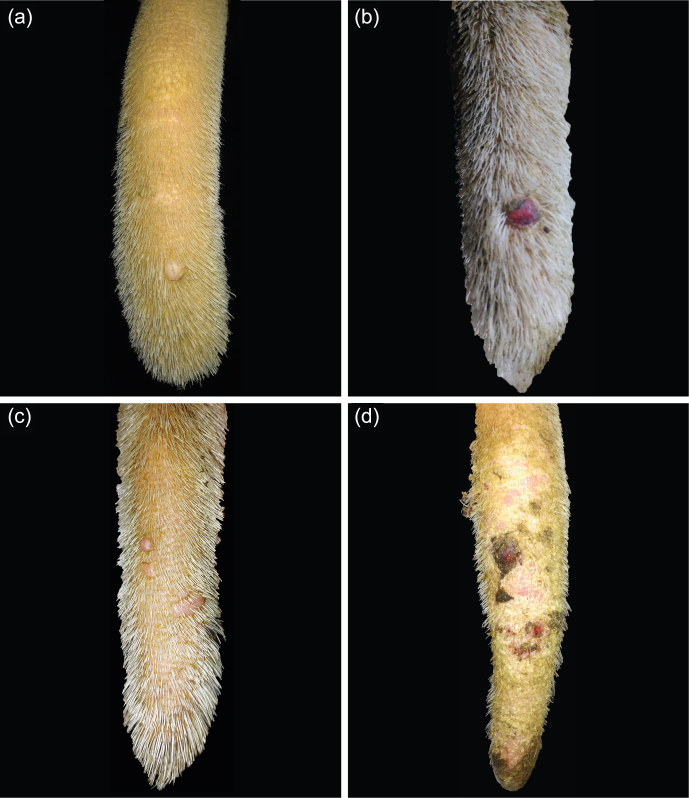
Lesion: fibroma. **(a)** Single, bland fibroma; **(b)** single, bruised fibroma; **(c)** multiple bland fibromas; **(d)** multiple bruised fibromas.

**Figure 7:**
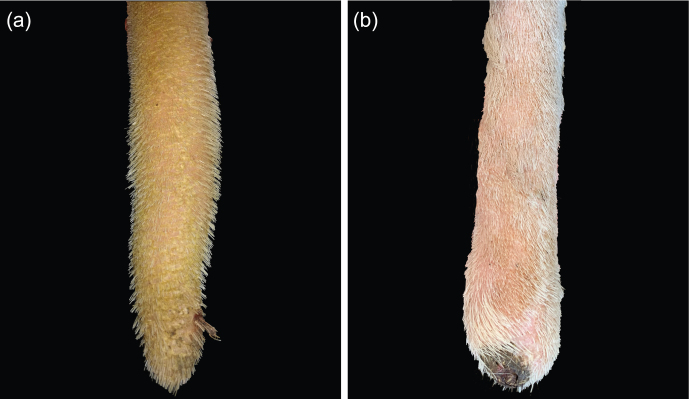
Lesion: swelling. **(a)** Slight swelling, **(b)** obvious swelling.

**Figure 8:**
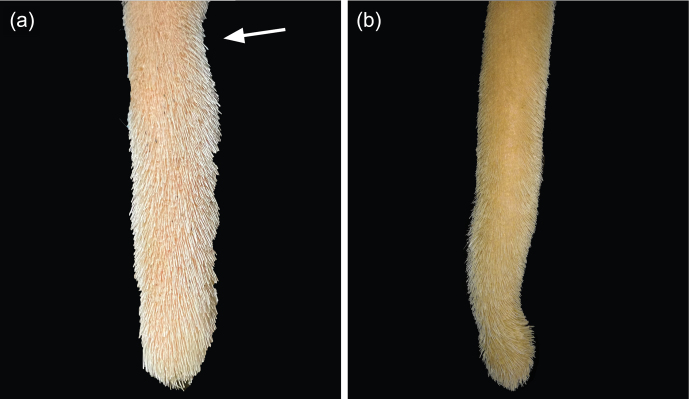
Lesion: thinning. **(a)** Narrowing of an upper part of the tail tip (arrow; always combined with a slight axis deviation). **(b)** Narrowing of a lower part of the tail tip.

**Figure 9:**
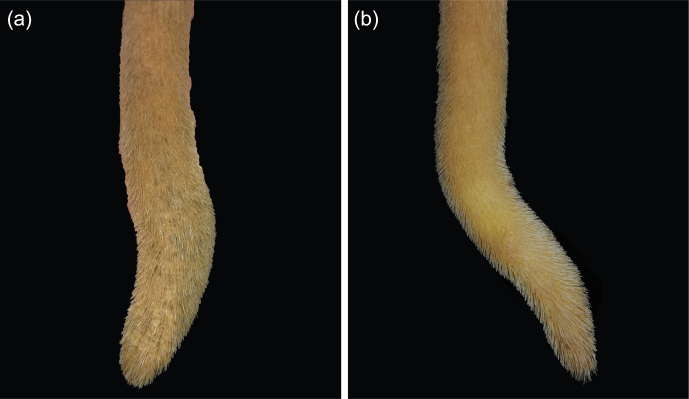
Lesion: axis deviation. **(a)** Wave-like, **(b)** angle-like.

After evaluating the whole data set, we clustered seven different kinds of lesions observed at the distal quarter of the tail (Table 2; Figs. 3–9). The different types of lesions and their manifestations were grouped and described as shown in Table 2.

**Table 2:** Description of the observed lesions, including their different manifestations.

Lesion	Manifestation			
Tail tip lesion (very tip of the tail; TT)	Hair loss	Hair loss + erythema and/or scab	Hair loss + bloody scab/bleeding	necrosis
Annular lesion (AN)	Hair loss	Hair loss + constriction	Hair loss + constriction + secretion/bleeding	Laceration/part loss
Hyperkeratosis (HK)	Small scales	Spruce-cone-like scales	Spruce-cone-like scales + fissures	
Swelling (SW)	Slight swelling, palpable as edema, barely visible	Obvious swelling, visible and palpable as edema		
Axis deviation (A)	Wave-like	Angle-like		
Thinning (T)	Thinning of a single part of the distal tail			
Fibroma (FB)	Single, bland	Multiple, bland or single, bruised	Multiple + bruised	

Lesions affecting the very tip of the tail (TT; Fig. 3) included hair loss, erythema, scabs, or necrosis. The distal part of the tail frequently showed one or more annular lesions (ANs; Fig. 4). ANs varied between hairless rings, annular constrictions, and bleeding rings or rings covered by wound secretion (partly encrusted). All kinds of ANs appeared to be either full circles or semicircles.

In addition, we observed hyperkeratosis (HK; Fig. 5) appearing as dry skin covered by small scales or spruce-cone-like scales, partly resulting in fissures and cracked skin. Moreover, we observed fibromas (FBs; Fig. 6), firstly described as verruca-like masses, which appeared either singly or multiply and as bland or bruised. Furthermore, we found swellings (SWs; Fig. 7) of the tail tips and categorized them as slight SWs if they were barely visible but palpable as edema or as obvious SWs if they were both palpable as edema and visible. Another finding was the thinning of tail tips (Fig. 8). In these cases, a short part of the tail tip did not follow the physiological tapering towards the tip. During palpation, thinnings differed from the remaining tail tip, being firmer and less cushioned. Most of the time, thinnings appeared in combination with axis deviations (A/T; Fig. 9). These deviations were slightly inflexible and were differentiated as being either wave-like or angle-like.

Based on those seven different clusters of findings, we prepared a training card, giving an overview of the observed tail tip lesions and their manifestations (Fig. 2).

### 3.2 Frequencies of tail tip lesions in dairy cows

In part two of the study, the frequency of any types of lesions in all examined tail records ( n=3587 ) was 90.2 % ( ±0.8 ). The frequencies of all occurring types of lesions per breed are shown in Table 3. The frequencies of all occurring types of lesions per herd are shown in Table 4.

**Table 3:** Least-square means ( ×100± standard errors of estimation) of frequencies of lesions in Holstein Friesian and Simmental cows considering n=3587 tail examinations originating from 641 cows.

Frequency of tail tip lesions	Holstein n=2669	Simmental n=918
All types of lesions, all herds (%)	87.7 ( ±0.9 )	98.0 ( ±1.6 )
Tail tip lesion (very tip of the tail), all herds (%)	27.8 ( ±1.6 )	71.6 ( ±2.8 )
Annular lesion, all herds (%)	26.7 ( ±1.7 )	28.6 ( ±3.2 )
Hyperkeratosis, all herds (%)	65.0 ( ±1.5 )	21.6 ( ±2.7 )
Swelling, all herds (%)	38.1 ( ±1.7 )	66.4 ( ±3.1 )
Axis deviation/thinning, all herds (%)	25.2 ( ±1.5 )	22.2 ( ±2.8 )
Fibroma, all herds (%)	9.6 ( ±1.1 )	20.7 ( ±1.9 )

**Table 4:** Least-square means ( ×100± standard errors of estimation) of frequencies of tail lesions in Holstein Friesian and Simmental cows ( n= 3587 tail examinations) by herd.

	Frequency of tail tip lesions per herd				
	Holstein Friesian			Simmental	
	Herd A n=573	Herd B n=1716	Herd C n=380	Herd D n=495	Herd E n=423
All types of lesions (%)	84.0 ( ±2.0 )	86.0 ( ±1.2 )	94.1 ( ±1.8 )	99.0 ( ±2.2 )	97.0 ( ±2.2 )
Tail tip lesion (very tip of	32.4 ( ±3.2 )	14.3 ( ±2.0 )	47.0 ( ±2.6 )	83.6 ( ±3.6 )	59.3 ( ±3.6 )
the tail; %)					
Annular lesion (%)	26.9 ( ±3.9 )	15.3 ( ±2.3 )	42.5 ( ±2.8 )	26.1 ( ±4.2 )	31.3 ( ±4.3 )
Hyperkeratosis (%)	39.5 ( ±3.2 )	68.8 ( ±1.9 )	75.1 ( ±2.7 )	7.4 ( ±3.5 )	35.8 ( ±3.5 )
Swelling (%)	12.1 ( ±2.9 )	19.8 ( ±1.7 )	82.2 ( ±2.5 )	56.0 ( ±3.1 )	77.1 ( ±3.1 )
Axis deviation/thinning (%)	5.2 ( ±3.1 )	16.0 ( ±1.8 )	50.4 ( ±2.4 )	9.4 ( ±3.4 )	35.3 ( ±3.4 )
Fibroma (%)	10.9 ( ±2.4 )	7.0 ( ±1.4 )	13.7 ( ±2.0 )	11.2 ( ±2.7 )	30.2 ( ±2.6 )

Additionally, for interested readers, the prevalences of the different, binary-encoded tail tip alterations at each time of data collection are given in the Appendix.

In part two of the study, we did not find any lesion that did not fit into the outline shown on the training card (Fig. 2). 

## 4 Discussion

The current study focused on three main questions regarding the existence, the different manifestations, and the frequency of tail tip lesions in dairy cows. For this purpose, n=3587 tail tip examinations originating from 641 different cows from five different German dairy herds were evaluated in total. Of these, 90.2 % ( ±0.8 ) had one or more lesions. Regarding the literature on lesions of the tail, fractures and docked tails are the most commonly found in dairy cows so far (Moono et al., 2022; Zurbrigg et al., 2005; Olsen et al., 2023). In the PraeRi study (Hoedemaker, 2022), a large German survey on cattle welfare, apparent fractures defined as axis deviations and tissue enlargements were recorded in German dairy cows, with reported prevalences of between 4.5 % and 15.7 % ( n=765 ). Moono et al. (2022) published the first quantitative study of the frequency of tail damage within New Zealand dairy farms. They surveyed prevalences of fractures of 2.1 %–13.2 % in different regions of New Zealand. Additionally, they quantified the prevalences of damaged and docked tails and concluded that the frequency of tail injuries among New Zealand dairy cows had increased over the 4-year period of data collection. Olsen et al. (2023) recently reported a prevalence of 45.8 % in tail fractures in US dairy cows, with an increase in multiparous cows and in cows with repeated mastitis treatments. However, all studies are based on different scoring systems and mainly focused on fractured or deviated tails. To our knowledge, the current study is the first to examine the spineless part of the tail in dairy cows, focusing on the different types of lesions that can occur and their frequency. It is important to note that this is a small sample and cannot be generalized as cows from only five different German herds were included in the study. However, the results showed high frequencies of tail tip lesions in all samples. Since tail tip lesions occurred even in small samples, we concluded that the observed lesions represent a remarkable problem in dairy cows. Even more so, because the tail tip is mostly covered by the hair of the switch, lesions in this region may often remain unnoticed (George et al., 1970).

During part one of the study, it became apparent that findings on the tail tips varied between examinations in terms of their manifestation and that lesions could appear or disappear within the examination interval. Therefore, we examined most of the cows repeatedly over a time period of 6–8 months. It must be taken into account that this could have led to an overestimation of the frequencies. We tried to reduce this bias by including the individual cow studied as a random effect in the statistical model. When analyzing the data, we also found out that we were unable to reliably distinguish whether a lesion had persisted or originated from a previously recorded one or whether the previous lesion had healed and a new one had emerged. Therefore, it was not possible to draw conclusions about lesion progression from this data set. However, future studies of lesion prevalence and progression are needed to make a reliable statement in this regard.

In the course of our study, it became obvious that cows suffer from more lesions beside fractures and axis deviations. The TT lesions showed frequencies of affected tail tip records of 27.8 % ( ±1.6 ) in HOL cows and 71.6 % ( ±2.8 ) in SIM cows. Furthermore, percentages of affected tail tip records regarding TT lesions varied from 14.3 % ( ±2.0 ) to 83.6 % ( ±3.6 ) between the different herds. The lesions found at the very tip of the tail included hair loss, erythema, scabs, bleeding, and necrosis. These symptoms are also described as an early stage or a mild case of tail tip inflammation and necrosis appearing in fattening bulls (Drolia et al., 1991; Dirksen et al., 2006), especially when housed intensively and on slatted floors (Schrader et al., 2001). The data structure of our study does not allow us to assess a possible correlation between the occurrence of lesions and the housing environment. However, poisoning resulting from, e.g., the intake of ergot alkaloid mycotoxins (Cowan, 2020; Rahimabadi et al., 2022) or Ramaria flavo-brunnescens, a mushroom common in South America (Scheid et al., 2022), and Degnala disease (Sikdar et al., 2000) are also described to lead to hairless, sore, or necrotic tail tips in male and female cattle.

Besides necrosis of the tail tips, we found signs of inflammation like erythema and swelling. Swellings were highly prevalent ( 38.1%±1.7 HOL, 66.4%±3.1 SIM; varying between herds from 12.1%±2.9 to 82.2%±2.5 ) and were always palpable as edema. Bony-callus formation in response to previous trauma can be ruled out as a cause of the swellings as the swellings were all localized at the spineless part of the tail. George et al. (1970) reported on necrosis of the tail in bovine animals, including male and female buffalo and cattle. According to this report, the onset of tail tip necrosis is characterized by slight swelling of the switch-covered part of the tail and therefore often remains unnoticed. During the further progression of the disease, the inflammatory symptoms extend upwards. The tail becomes insensitive and cold to the touch, and hair loss and a line of demarcation appear. Rahimabadi et al. (2022) describe an enlargement of the upper part of the tail, representing a symptom of ergotism after part loss of the distal part of the tail. Drolia et al. (1991) found histopathological evidence of edema in the tails of fattening cattle affected by tail inflammation and necrosis.

Another symptom described in the literature as an early symptom of tail tip inflammation and necrosis in fattening bulls is hyperkeratosis (Dirksen et al., 2006), which has also been confirmed histopathologically in the affected tails of fattening cattle (Drolia et al., 1991). The frequency of HK found in the present study varied between herds – from 7.4%±3.5 to 75.1%±2.7 – and between breeds ( 65.0%±1.5 HOL, 21.6%±2.7 SIM), indicating that hyperkeratosis can appear in high or low frequencies and does not represent a physiological state of the tail tip. Drolia et al. (1991) noted that hyperkeratosis and crusting represent mild cases rather than early stages of tail tip necrosis. Histologically, these symptoms were accompanied by intramural and perivascular edema and hemorrhages. Based on the histological findings, the authors concluded that tail tip necrosis might be the result of cutaneous ischemia, which finally results in tail tip inflammation and necrosis.

In the current study, we observed ANs that appeared to be fully circular or semicircular. Annular constrictions have also been described in the literature as a symptom of ischemia caused by disturbed blood flow due to vasoactive substances, e.g., mycotoxins, circulating in the blood vessels; this is even more so the case in the fine acral blood vessels of the tail (Cowan, 2020; Rahimabadi et al., 2022; Scheid et al., 2022). We defined annular hair loss, annular constriction, bleeding rings, and annular constrictions resulting in the loss of the distal part of the tail as one group of lesions. Frequencies of ANs varied between herds from 15.3%±2.3 to 42.5%±2.8 and between breeds from 26.7%±1.7 in HOL to 28.6%±3.2 in SIM. Different manifestations occurred, and examiners reported ANs as a recurring lesion. As mentioned above, George et al. (1970) also described a visible line of demarcation in the course of tail tip necrosis and found clotted blood within the coccygeal vessels, obliterating blood supply. In order to confirm their hypothesis that tail tip necrosis was a result of ischemia, the authors attempted to treat the early stages – represented by hair loss, insensitivity, and being cold to the touch – with a parasympathomimetic drug administered locally by injection into the tail. This treatment resulted in healing due to improved blood flow following vasodilation.

Further lesions observed at the tail tip were A/T and FBs. Regarding both, we could not find any reports in the literature, although pictures of tail tip necroses where A/T can be seen are published, but this is not described as a symptom (Drolia et al., 1991). In our study, the percentage of affected cows with A/T varied from 5.2%±3.1 to 50.4%±2.4 between herds and varied between breeds from 22.2%±2.8 in SIM to 25.2%±1.5 in HOL. These findings do not support a hypothesis of sporadic occurrence; therefore, etiology and occurrence should be investigated in future studies. During data collection, FBs were firstly described as warts; however, histopathological examination revealed fibromas (unpublished data). A fibroma is known as a benign neoplasm of the fibroblasts and collagen matrix, most often seen in the dermis or subcutaneous tissue. It is not very common in cattle. It may be associated with bovine papilloma virus and/or trauma (Queiroz et al., 2022). The percentage of affected cows with FBs in the current study ranged between herds from 7.0%±1.4 to 30.2%±2.6 and between breeds from 9.6%±1.1 in HOL to 20.7%±1.9 in SIM; thus, FBs were least prevalent. Cows affected by FBs at the tail tip showed either single or multiple bland or bruised neoplasms, which also differed in size. Examiners reported diameters between approximately 0.3–2.0 cm. In the literature, no reports were found regarding the meaning of a fibroma as a lesion of the tail tip. As trauma can cause the onset of fibromas (Queiroz et al., 2022), FBs should also be further investigated in order to clarify if a causal relationship with the occurrence of tail tip necrosis does exist.

In this study, we found a high frequency of different tail tip lesions in all flocks, although they were kept in different housing systems (Table 1), and in both breeds studied. According to Muley et al. (2016), three organ systems are particularly susceptible to the development of inflammatory pain: the skin, the joints, and the gut. Most of the recorded lesions affect the skin. It is therefore reasonable to assume that the lesions are also associated with pain. It has also been reported that tail tip lesions in cattle can lead to bacteraemia and death (Thomson et al., 2009). Thus, our results might indicate a serious animal welfare issue as the alterations either represent painful lesions or can result in severe complications. Our research included five different farms which are all located in Germany. In order to verify if tail tip lesions in dairy cows are an unnoticed yet highly prevalent problem, future studies should examine additional farms in different countries and regions.

Taken together, the high frequency of tail tip lesions is an alarming signal that cows could be eminently affected by inflammation of the tail while, most often, the lesions may remain unnoticed due to the hair of the switch.

The literature regarding examinations of tail tip inflammation and necrosis in dairy cattle is scarce, and, as a result, the pathogenesis remains unclear (George et al., 1970; Ural et al., 2007; Heers et al., 2017). Most often, tail tip necrosis is reported to be an issue in fattening cattle (Drolia et al., 1991; Schrader et al., 2001; Madsen and Nielsen, 1985). It is associated with slatted flooring systems (Schrader et al., 2001; Madsen and Nielsen, 1985; Drolia et al., 1990) or feeding high-energy ratios (Kordowitzki, 2015) as subacute ruminal acidosis triggers the onset of systemic inflammation, especially within the acra (Abdela, 2016). However, tail tip inflammation is reported to appear in many other species as well, such as buffalo (Barakat et al., 1960), rodents (Crippa et al., 2000; Recordati et al., 2015), cats (Bridle and Littlewood, 1998), deer (Ferguson et al., 2016), or pigs (Reiner et al., 2021). The consensus for all species regarding tail lesions is that the pathogenesis is not fully understood yet. The etiology of tail inflammation seems to be manifold. Ringtail in rats appears as an annular constriction, often followed by hemorrhages and finally dry necrosis of the distal part of the tail. Its etiology is influenced by low environmental humidity ( <25% ), although further influencing factors such as dietary deficiencies, genetics, environmental temperature, and the degree of hydration of the animal are discussed. Besides the annular constriction of the tails, epidermal hyperplasia with orthokeratotic or parakeratotic hyperkeratosis is described (Barthold et al., 2016). Recent studies regarding tail inflammation in pigs focused on the description of symptoms and the reasons for onset (Reiner et al., 2021; Ringseis et al., 2021; Kuehling et al., 2021). SINS (Reiner et al., 2021) has been defined as affecting tails, ears, claws, teats, and faces. The prevalence of SINS is very high ( >80% ) (Kuehling et al., 2021; Reiner et al., 2019). SINS has an endogenous etiology, and the symptoms mirror a systemic inflammation at outer body parts (Ringseis et al., 2021). Actually, there is no evidence that a bovine inflammation and necrosis syndrome comparable to SINS in pigs might exist. However, the types of lesions found during this study and the high frequencies oblige us to investigate if shaved, undocked tails reveal a so-far-unnoticed animal welfare issue in dairy cows which also mirrors systemic inflammation due to endogenous triggers. The developed training card seemed to be helpful as no lesions were found in part two of the study that did not match the lesions depicted. The further development of the training card into a scoring scheme should also be considered. A scoring scheme for tail lesions in dairy cows, as has already been developed for pigs (Valros et al., 2020), could be useful in assessing animal welfare.

Even though we investigated only a small sample of cows, we consider it to be very unlikely that all of the lesions observed were caused by the housing system, scrapers, rotating brushes, or treads from other animals. The lesions we observed did not resemble traumatic lesions, as would be expected from contusions. However, further studies are needed to refute the hypothesis of mechanical irritation or trauma as the sole cause of tail tip lesions. The symptoms are more similar to those of impaired blood circulation and ischemia, as described in the literature for many species. Finally, it should be noted that tail tip lesions in dairy cows merit further investigation as their etiology, their histopathology, and their course are unknown to date. In case the surveyed lesions also mirror systemic inflammation, as described in pigs (Reiner et al., 2021; Kuehling et al., 2021), tail tip scoring in dairy cows could be helpful in order to assess herd health and to improve animal health and welfare.

## 5 Conclusions

The dairy cows examined during the course of this pilot study displayed a wide variety of tail tip lesions. All of the observed lesions were summarized and categorized, resulting in seven different groups. The high frequencies of the lesions detected in this study throughout the different herds and breeds might indicate a notable animal welfare issue, and further research is needed to elucidate their etiology, pathogenesis, and risk factors. 

## Data Availability

No data sets were used in this article.

## Appendix A

**Table A1:** Prevalences ${}^{1}$ of the different tail tip lesions, binary encoded, at each data collection point per herd.

	Data collection									
	1	2	3	4	5	6	7	8	9	10
Tail tip lesion										
Herd A (%)	27.6	19.0	22.9	20.8	27.3	34.0	35.0	45.6	38.6	
Herd B (%)	10.4	6.5	13.6	17.8	10.7	5.6	19.4	21.2		
Herd C (%)	50.0	61.5	52.9	53.1	43.5	48.0	73.3	27.3	48.4	50.0
Herd D (%)	75.0	77.4	93.6	91.2	83.7	82.8	69.4	86.0		
Herd E (%)	45.9	51.6	38.7	61.3	75.8	74.2	66.1	58.1		
Annular lesion										
Herd A (%)	27.6	27.6	26.6	24.7	25.5	22.6	26.7	29.8	26.8	
Herd B (%)	7.3	8.9	8.5	11.1	16.8	15.2	18.4	23.9		
Herd C (%)	40,9	69.2	51.0	42.9	43.5	34.7	46.7	45.5	45.2	35.0
Herd D (%)	20.0	26.4	29.8	33.3	30.6	27.6	28.6	28.0		
Herd E (%)	27.9	19.4	22.6	30.6	38.7	35.5	33.9	43.5		
Hyperkeratosis										
Herd A (%)	31.0	27.6	29.4	35.8	47.3	56.6	51.7	40.4	33.9	
Herd B (%)	55.2	65.0	67.6	66.7	80.6	79.8	66.8	67.6		
Herd C (%)	28.6	50.0	66.0	85.4	91.3	98.0	93.3	90.9	67.7	85.0
Herd D (%)	3.3	3.8	4.3	8.8	6.1	12.1	10.2	12.0		
Herd E (%)	21.3	35.5	17.7	19.4	37.1	38.7	69.4	48.4		
Swelling										
Herd A (%)	8.6	8.6	9.2	9.4	16.4	15.1	18.3	17.5	14.0	
Herd B (%)	9.4	14.5	24.4	22.7	13.8	22.2	27.6	19.6		
Herd C (%)	63,6	69.2	78.4	79.6	76.1	96.0	86.7	90.9	93.5	90.0
Herd D (%)	35.0	54.7	66.0	66.7	71.4	67.2	40.8	60.0		
Herd E (%)	41.0	54.8	74.2	80.6	91.9	88.7	96.8	87.1		
Axis deviation/thinning										
Herd A (%)	5.2	5.2	5.5	5.7	7.3	5.7	3.3	3.5	5.4	
Herd B (%)	9.4	9.8	9.4	12.1	12.8	21.2	18.4	25.7		
Herd C (%)	45.5	69.2	37.3	49.0	54.3	46.2	53.3	50.0	54.8	60.0
Herd D (%)	10.0	7.9	10.6	8.8	10.2	8.6	6.1	10.0		
Herd E (%)	18.0	21.0	16.1	33.9	45.2	46.8	53.2	48.4		
Fibroma										
Herd A (%)	12.1	12.1	11.0	15.1	12.7	13.2	11.7	10.5	12.5	
Herd B (%)	6.3	6.1	7.0	12.6	8.7	8.2	5.1	4.2		
Herd C (%)	4.5	3.8	17.6	24.5	17.8	20.0	13.3	22.7	6.5	15.8
Herd D (%)	11.7	9.4	6.4	8.8	12.2	13.8	10.2	16.0		
Herd E (%)	13.1	17.7	17.7	29.0	48.4	45.2	32.2	35.5		

${}^{1}$ The prop.table function from the R basic package was used to determine the prevalence P of the different lesions. The results are based on the equation $P_{a}=n_{a}/n_{g}$ , where $P_{a}$ is the prevalence of a specific lesion; $n_{a}$ is the number of observed alterations, binary encoded; and $n_{g}$ is the total number of individuals observed.
